# Learned adaptive properties for mitigation of weight perturbations in embedded spiking networks

**DOI:** 10.3389/fnins.2026.1766765

**Published:** 2026-03-11

**Authors:** Sarah Luca, T. Patrick Xiao, Frances S. Chance, Sapan Agarwal, Corinne Teeter, G. William Chapman

**Affiliations:** 1 Sandia National Laboratories, Albuquerque, NM, United States; 2 Department of Mathematics, The University of Arizona, Tucson, AZ, United States

**Keywords:** context modulation, machine learning, neural-inspired computing, recurrent neural networks, spiking neural networks

## Abstract

Recent years have seen an increased importance of neural network inference in edge-based scenarios, which impose size and power constraints requiring novel computing devices. These same edge scenarios may require operating over long periods of time, or exposure to extreme environments, resulting in a drift of neural network weights that cause degraded performance. In searching for ways to develop neural network approaches that perform robustly under these conditions, we propose a biologically-inspired mechanism for the dynamic adaptation of within-neuron parameters that is guided by a global context signal carrying information about perturbations and variability in incoming stimuli. Specifically, we demonstrate that adaptive voltage thresholds or neuronal time constants, when informed by a global context signal, can enable network-level mechanisms to recover from perturbed synaptic weights. Consistent with prior literature, the context-modulated approach is effective for recurrent, but not feedforward networks, by modulating network level dynamics. We demonstrate this approach successfully recovers performance in image classification tasks and spatiotemporal tracking tasks under idealized and Gaussian noise as well as for realistic perturbations from a memristive device when exposed to ionizing radiation. Finally, we discuss how this approach enables the design of robust and energy-efficient neuromorphic systems that perform well, even in resource-constrained scenarios with extreme environments such as edge processing.

## 1 Introduction

In edge computing, computation is placed near sensors to perform embedded sensing-and-compute, to reduce data transmission to nearby processors, or to adhere to size-weight-and-power constraints. Neuromorphic processing, which integrates algorithmic insights from biological nervous systems into the hardware design, has emerged as a method to improve computational performance while adhering to these constraints. Such approaches often include analog computational elements, such as resistive crossbars, along with application-specific circuits to implement portions of neural network operations. Neural networks are good candidates for analog computation, because they are algorithmically robust to certain forms of non-idealities, such as small weight perturbations, quantized weights, and quantized activations (Rasch et al., 2023).

Analog components are often employed for specific sub-operations, such as matrix-vector multiplication, for their decreased energy consumption and hardware footprint compared to digital implementations. One of the most common approaches to analog neuromorphic networks is to implement the multiply-accumulate (MAC) operations of weighted activity as analog matrix-vector multiplication. These approaches use devices such as resistive memory (ReRAM) (Xia and Yang, 2019; Wan et al., 2022), floating-gate memory (Hasler, 2020), or capacitive memory (Kim et al., 2023) to implement activity-weight multiplication in the physical behavior of devices. In these cases, synaptic weight values are represented by the conductivity or the capacitance of the devices, although the underlying physics of the devices varies.

However, analog components are vulnerable to perturbations such as noise and drift, which generally worsen over time and under the extreme environmental conditions characteristic of many edge-computing scenarios. To mitigate these vulnerabilities, we propose a hardware-algorithm codesign strategy that introduces perturbation-adaptive parameters into neural network processing on analog devices. While environmental sources are likely to affect components that implement both weights and neurons, previous work (Xiao et al., 2025) has demonstrated that weight perturbation is the limiting factor in realistic scenarios. Thus our experimental approach assumes that environmental effects are captured entirely in weight perturbations, but the adaptive dynamics that we propose could likely mitigate well-modeled effects on neural properties as well.

**Figure 1:**
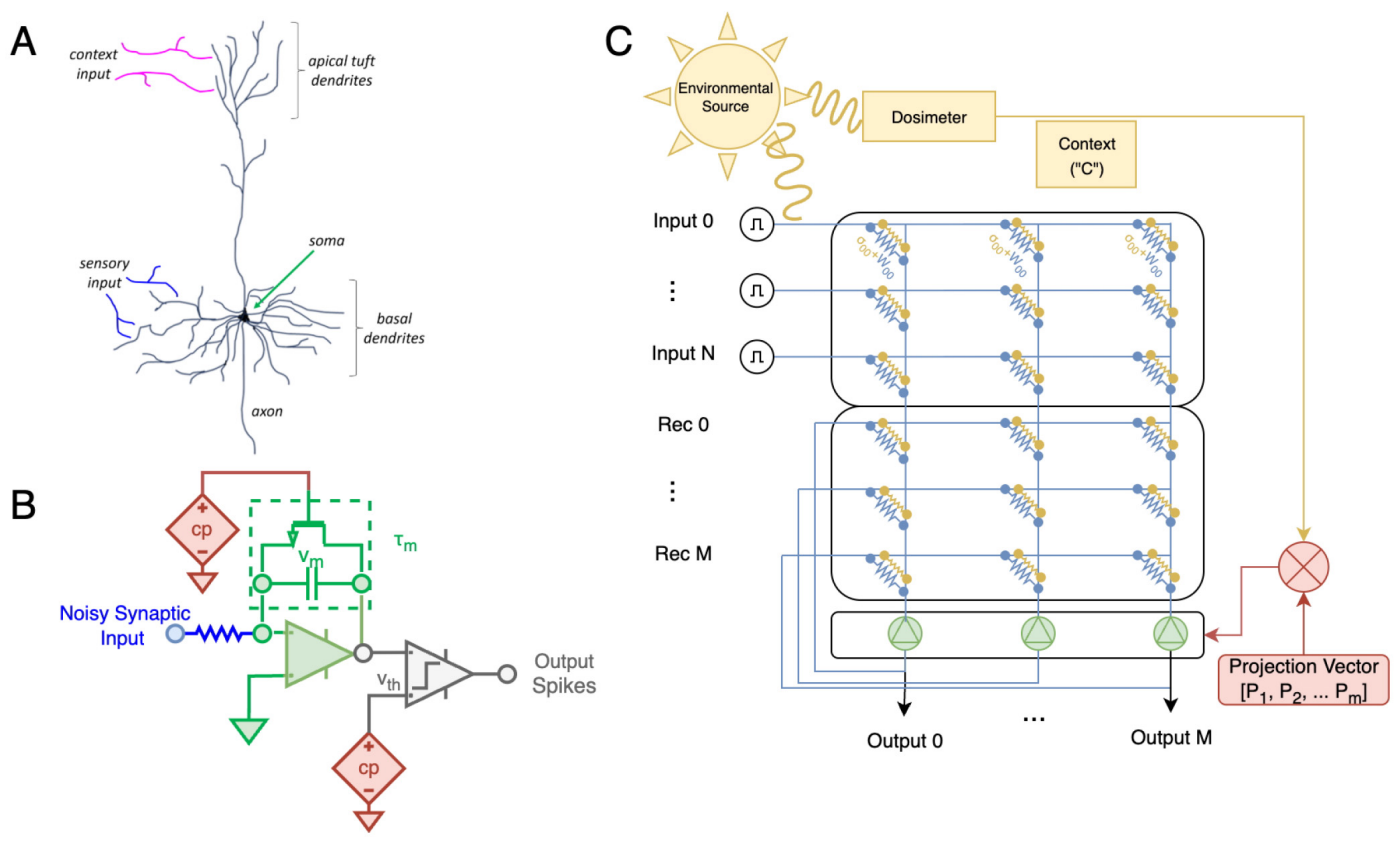
Overview of biologically inspired context signals, from neuroscience, to individual circuits, and system-level design. **(A)** An illustration of a pyramidal neuron with apical tuft dendrites and basal dendrites for integration of top down (context, magenta) and bottom up (sensory, blue) information. The sensory and contextual signals both affect the dynamics of the soma (green) and output activity through the axon (gray). This structured input of sensory and context inputs is the basis of our approach. **(B)** A simple circuit implementation of the pyramidal neuron.Synaptic input results in a voltage accumulation across the central capacitor, and spikes are generated by comparing to *v*_*th*_ The context signal (“c," components in red) may modulate *either* the time constant of the soma through the transistor, or may change the threshold. **(C)** Example configuration of an entire system. An environmental source of degradation (e.g., radiation, yellow) effects analog-devices encoding weights (blue), introducing perturbed (yellow weights) in a neural network, while an auxiliary device (e.g., dosimeter) measures lifetime exposure to this source of perturbation. Weight perturbations are illustrated as a second set of elements (yellow resistors) to emphasize that they constitute a separate physical process from the ideal weights, but in practice they occur within the same devices (blue resistors). While weight perturbation increases in response to the environmental source, total current flowing into analog electronic neurons (green) begins to shift from ideal. The context signal is multiplied by a *per-neuron* adaptive parameter to modulate intracellular processing, such as firing threshold or time constant.

Our overall approach leverages a *context signal*, which is informative of the overall degree of perturbations affecting the analog devices at a particular point in time. This context signal is received by individual neurons to dynamically change their properties, so that they produce a corrected population-level activity in response to perturbations to the synaptic weights (see Figure 1C), resulting in improved task performance.

### 1.1 Prior work

We broadly classify prior work on improving analog neural networks into two categories: sub-algorithmic and algorithmic. Sub-algorithmic approaches focus on reducing hardware nonidealities to improve the fidelity of low-level operations such as matrix-vector multiplication. Algorithmic approaches, by contrast, explicitly model and optimize hardware for data-driven computation, such as through hardware-aware training.

#### 1.1.1 Sub-algorithmic approaches

To further increase the robustness of neural networks to weight perturbations, changes can be made to either the MAC or activation sub-algorithms of neural network layers. One common approach is to quantize analog weights, such that slight perturbations in device parameters do not result in a difference in downstream MAC activity (Ma et al., 2023; Hubara et al., 2017). This approach is effective at low levels of perturbation, but with continued exposure, such as ionizing radiation, errors accumulate over time and result in overlapping weight distributions which are both mean-shifted and higher in variance (Xiao et al., 2024). More complex approaches involve spreading weight representations across more devices to increase the effective precision of their combination. Mean-shifts in conductivity can be corrected by altering the activity of analog-to-digital converters, but at the cost of increased complexity (Xiao et al., 2025). Overall, modification of the sub-algorithms required for neural network inference can mitigate low levels of perturbation, but decrease overall performance of the network through quantization, or increase hardware footprints.

#### 1.1.2 Algorithmic approaches

Whereas sub-algorithmic approaches aim to increase the ideality of sub-operations, algorithmic approaches use the hardware behavior in one form or another to change the overall network operations. For instance, hardware aware training (HAT) (Momeni et al., 2025; Wright et al., 2022) utilizes realistic simulations of device behavior during training, in combination with approaches such as surrogate gradient descent (Klachko et al., 2019; Rasch et al., 2023). Such approaches can fit non-linearities, hysteresis effects, and noise of underlying hardware, and therefore represent a form of physics informed neural network (PINN) (Raissi et al., 2019), rather than typical linear weights and non-linear activation functions. HAT has been used for both ANN and SNN mitigation of noisy parameters, by training with noisy weights to mitigate a given level of noise (Falk et al., 2025; Khan et al., 2023).

Other approaches such as *in-silico* or hardware-in-the-loop training allow training on analog hardware, rather than the *a priori* simulation of HAT (Laydevant et al., 2024; Zhang and Sitte, 2006). While effective, this strategy requires per-device training, a method for implementing either backpropagation (Renner et al., 2021) or local learning rules (Falk et al., 2025), and continual learning (Kudithipudi et al., 2022). Such approaches are therefore cumbersome and may require continual rewriting of weights to mitigate effects of noise, which can be a slow or energetically consumptive process, and increase the hardware footprint.

### 1.2 Current work

#### 1.2.1 Perturbation mitigation: adaptive parameters

Both the algorithmic and sub-algorithmic approaches discussed above implement a form of static mitigation, by modifying device operation or network operation to tolerate a specifically chosen level of perturbation. To operate reliably under dynamically changing levels of perturbation, other mitigation strategies are needed. We explore whether these errors can be compensated by tunable neuron-specific components whose functionality adapts in response to a “context" signal that is related to the changing level of synaptic degradation, without requiring direct modification to the synaptic weights. In contrast to the weights, which are encoded in non-volatile memory devices, we seek a method to utilize other circuitry in analog neural networks, which is controllable at run-time, to modify network dynamics in response to the context signal. One common method of employing ReRAM in neural networks is to combine the resistive array, implementing the weights, with complementary metal-oxide-semiconductor (CMOS) circuits which implement a spiking neural network (SNN) (Li et al., 2025). In this arrangement the “soma” of the spiking neuron functionally replaces the analog-to-digital converter with a high-efficiency spiking mechanism (Figure 1B). While previous work utilizes biasing voltages to set parameters such as time-constant or threshold voltage, they have not combined these tuneable parameters with dynamic control mechanisms. We therefore aimed to design context-dependent, adaptive parameters in simple spiking neural network (SNN) architectures, and evaluate whether the network itself can learn to use these parameters in combination with the context signal to mitigate degraded synaptic weights.

#### 1.2.2 Biological motivation for context

In experimental neuroscience, the concept of “context” has been used as a global, top-down, signal which measures large-scale certainty or identity of information. The interaction of such contextual information with sensory-driven input has been implicated in multi-tasking and continual learning (Rangel et al., 2014) by reducing the interference that can occur when learning a new task (Aimone and Severa, 2017), which otherwise leads to catastrophic forgetting of previously learned tasks. Biological studies have shown that the brain integrates top-down contextual information with bottom-up sensory input in neocortical neurons (Gilbert and Li, 2013). In particular, pyramidal neocortical neurons have been implicated in this form of computation based on their morphology, which allows for the integration of two electrically-segregated information streams: a distal top-down signal (e.g., via the apical tuft) and a proximal bottom-up signal (Figure 1A) (Schuman et al., 2021). Computational models have shown that such pyramidal neurons, endowed with synaptic plasticity that utilizes contextual information, are able to perform context-dependent processing (Baronig and Legenstein, 2023).

Distal modulation through apical dendritic inputs has been shown to have a multiplicative effect on proximal inputs (Ferrand et al., 2023). Other studies have demonstrated that context-based processing can be achieved through simple spike-threshold modulation in liquid state machines (Helfer et al., 2023). These prior results suggest that context signals that non-linearly alter the response properties of neurons may provide a mechanism for altering network activity in response to a simple feedback signal.

#### 1.2.3 Contributions

Here, we investigate the use of a context signal that measures the global degree of perturbation to network weights to modify the behavior of simple leaky-integrate and fire neurons. This is achieved through a learned parameter that controls either the firing threshold or the membrane time constant of the neuron, in proportion to the overall context signal. By demonstrating efficacy across multiple adaptive parameters, we demonstrate that the exact mechanism of the context-dependent adaptation is not essential, and a neuron may in fact incorporate multiple adaptation mechanisms. We demonstrate our algorithmic approach across two tasks; a static image classification task, and a temporal object-tracking task. We also test on two sources of network error; an idealized Gaussian distribution, and the other based on data collected from a real resistive crossbar. Across the combination of perturbation sources and tasks, we show that context-dependent adaptations, both in the form of threshold and time-constant modulation, are able to support high-accuracy recurrent neural network operation in the presence of a high degree of perturbation. Through further investigation of the distribution of learned adaptive parameters and network-level dynamics, we illustrate how recurrent networks utilize these adaptive parameters, while feedforward networks are unable to.

Overall, our results show that global context signals, corresponding to the degree of perturbation, can be used with neuron-specific learning of adaptive parameters to overcome the network effects of synaptic weight perturbations. These methods support theoretical frameworks for perturbation-tolerant processing in biological systems, and open the pathway for efficient and adaptive processing in edge-based neuromorphic platforms.

## 2 Methods

Our overall methodological approach is visualized in Figure 1C. We train neural networks implemented with analog synaptic weights with independent and identically distributed (IID) perturbations added on a per-weight basis up to a known maximum level of perturbation intensity. The source of the perturbation also informs a global “context” signal, which is available to the spiking activation function. Each unit in the spiking layer has a trainable parameter that uses the context signal to change either the voltage threshold or the membrane time constant. This parameter is multiplied by a global scalar value which is provided by a measurement device that provides a measure of the overall exposure to sources of weight perturbation, such as a dosimeter, thermometer, or clock. The network dynamics are therefore dynamically adaptive to the same environmental sources which perturb analog weights, and adapt in real time during deployment.

### 2.1 Network architecture and dynamics

**Figure 2:**
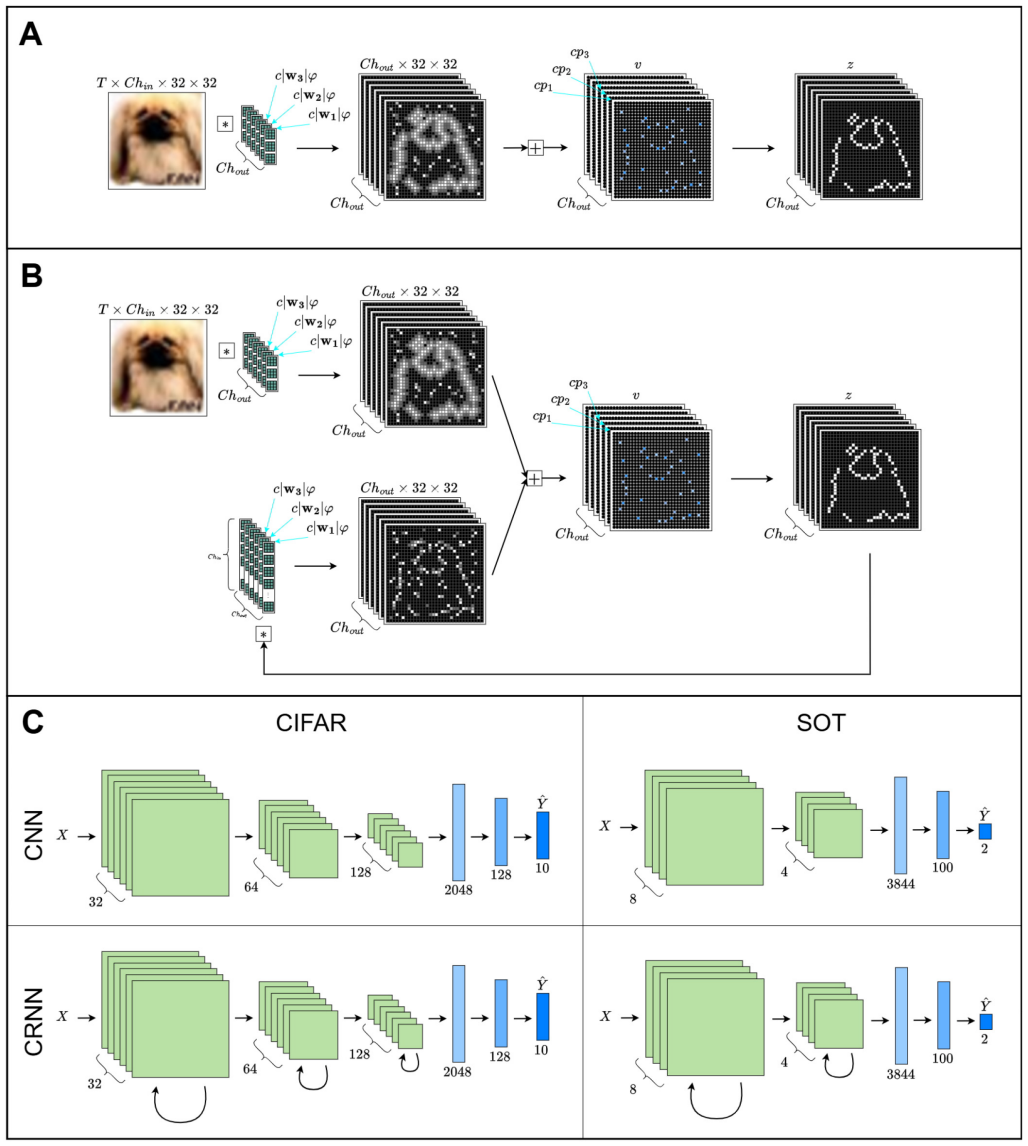
Network diagram. **(A)** The feedforward modules convolve the input with the weights, which have perturbations added proportionally to them and the context input (see Equation 6). The context neurons learn the parameter **p** which adjusts either the voltage threshold or the time-constant for each channel based on the context input (see Equations 4, 5), modulating the firing rate of the neuron. **(B)** Same as **(A)**, but showing the additional recurrent convolutional weights. **(C)** Block diagrams for each network type and dataset. Each green block represents a module from either **(A)** or **(B)**, resulting in a decreasing spatial resolution but increasing number of channels. After the final green block, there is a linear readout by flattening spatial dimensions and utilizing a leaky-integrator to accumulate spikes through time (blue rectangles).

We utilize two network architectures, a feedforward and recurrent version, both of which are based on standard convolutional network architectures from the machine learning literature. The feedforward network includes three VGG-like (Simonyan and Zisserman, 2015; Sengupta et al., 2019) modules of a convolution layer followed by batch normalization, the activation layer composed of LIF neurons, and max pooling (Figure 2A). The recurrent network has a similar network architecture with convolutional recurrent (CRNN) layers (Ballas et al., 2016), which have a second set of convolutional kernels that take the spiking activity on each timestep and introduce them as a separate stream of inputs (Figure 2B). The CRNN layers do not utilize batch normalization in order to maintain the hardware benefits of having a binarized activation function. Both architectures have a final module for flattening the feature space (Figure 2C) for the final classification layer. The activation function of the last module is a leaky-integrator (LI), rather than a spiking activation, to allow accumulation of spikes over a trial. Stimuli, in the form of frames as described in the exemplar descriptions below, are encoded as voltage injections to neurons in the first layer of the network.

### 2.2 Neural dynamics

#### 2.2.1 Leaky-integrate-and-fire (LIF)

We implemented spiking neural networks (SNNs) in Norse (Pehle and Pedersen, 2021) using the standard leaky-integrate-and-fire (LIF) model neuron dynamics; neuron parameters and descriptions can be found in Table 1. The membrane potential *v*_*j*_(*t*) of a LIF neuron *j* is described by the differential equation:


$$\begin{array}{l} \tau_{\text{m}}\frac{dv_{j}}{dt}=v_{\text{leak}}-v_{j}+I \end{array}$$ (1)

where *I*(*t*) is the input current, comprised as the weighted sum of presynaptic spikes through a weight matrix **w**, a voltage-encoded stimulus, or both. The parameter *v*_leak_ is the voltage leak at each time step (which is 0 for our experiments), and τ_m_ is the membrane time constant. We chose different values for τ_m_ in the feedforward and recurrent networks as described in Section 2.6.2. A “spike” (*z*_*j*_) is described by the jump condition


$$\begin{array}{l} z_{j}=\text{Θ}\left(v_{j}-v_{\text{th}}\right) \end{array}$$ (2)

where *v*_th_ is the spiking threshold defaulting to 1.0 (but see Section 2.2.2). After a spike, the membrane potential resets based on the transition equation


$$\begin{array}{l} v_{j}=\left(1-z_{j}\right)v_{j}+zv_{\text{reset}} \end{array}$$ (3)

where *v*_reset_ is the reset voltage of the neuron.

**Table 1:** All model variables, parameters, and descriptions.

**Group**	**Symbol**	**Description**	**Notes**
Stateful variables	*v*	Membrane potential	
	*I*	Input current to neuron	
	*z*	Spiking activity	
Functions	Θ	Heaviside activation	
Parameters	*v*_leak_	Leak voltage	0.0 V
	*v*_reset_	Reset voltage	0.0 V
	*v*_th_	Threshold voltage	
	τ_m_	Time constant	
	*v*_th_base__	Base threshold	1.0 V
	τ_m_base__	Base time constant	Hyper-parameter (FF: 4 frames, Rec: 16 frames)
Weight perturbations	*w*	Synaptic weight	Trainable parameter
	*w*_perturbed_	Perturbed synaptic weight	
	μ	Mean	
	σ	Standard deviation	
	*N* (μσ)	Gaussian distribution	
	φ	Random variable	
Context-modulation	*c*	Context	Trial-level singleton [0, 1.0]
	*p*	Context-dependent projection	Trainable parameter

#### 2.2.2 Context-dependent adaptation

The context-dependent adaptation modifies the dynamics of Equation 1 by changing *v*_th_ (adaptive threshold) or τ_m_ (adaptive time-constant) as a function of the context signal. Note that τ_m_ is different for each neuron *j* (τ_m_*j*__) for the adaptive time-constant experiments, but constant for the adaptive threshold experiments. Similarly, *v*_th_ is different for each neuron *j* (*v*_th_*j*__) in the adaptive threshold experiments, but constant for the adaptive time-constant experiments. In both cases, the value for each neuron is modulated by a trainable parameter *p*_*j*_ that is proportional to the context signal *c*. For the adaptive threshold, each *v*_th_*j*__∈***v*_*th*_** of Equation 1 (where ***v*_*th*_** is a vector of threshold values for each neuron in the layer) is modified by a context-dependent additive (Helfer et al., 2023) term *p*_*j*_*c*. Thus for all neurons in the layer, the threshold vector ***v*_*th*_** is described by Equation 4:


$$v_{th}=v_{th_{base}}+pc$$ (4)

where *p*_*j*_∈**p**. Note that each threshold in ***v*_*thbase*_** is the same for all neurons in the layer, with a default value of 1. We initialize each *p*_*j*_ according to a random normal distribution. Similarly, for the adaptive time-constant, each τ_m_*j*__∈τ_**m**_ is modified by the same additive term as the adaptive voltage threshold. Thus, the time-constant for all neurons in a layer τ_**m**_ is described by Equation 5:


$$\tau_{m}=\tau_{m_{base}}+pc.$$ (5)

**Figure 3:**
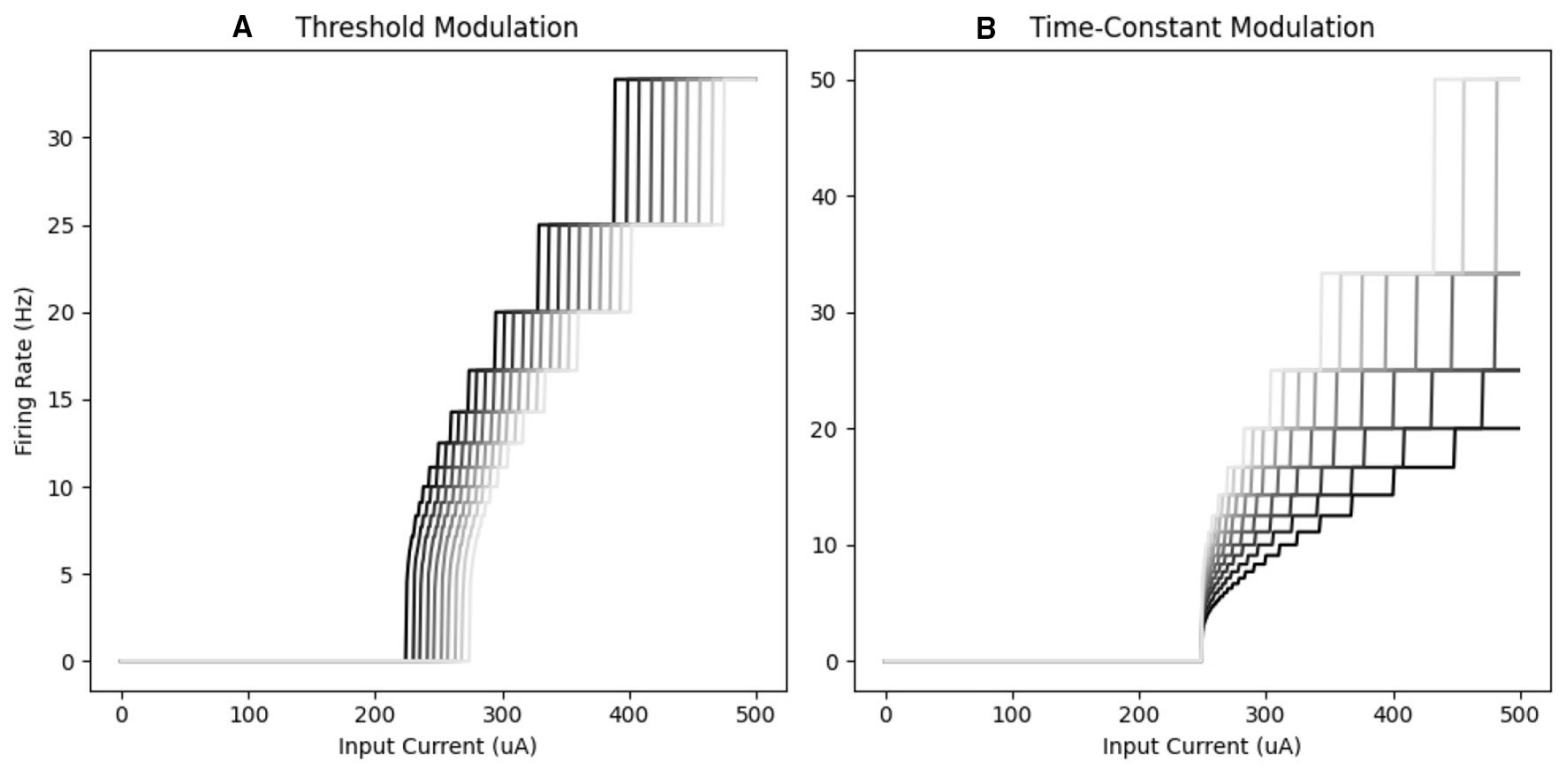
Schematic of the effects of context at the level of individual units. **(A)** The effect of a variable threshold on the FI-curve of an individual unit. Decreasing threshold (illustrated by darker curves) lowers mean activity (indicated by the leftward shift of FI-curves). **(B)** Increasing the time constant of an individual unit (illustrated with darker colors) resulted in a non-linear change in the FI curve, demonstrating that time constant modulation cannot be described as a change in bias alone.

As with the adaptive voltage threshold, each time-constant in τ_***m***_***base*** is the same for all neurons in the layer. The choice of this value is detailed in Section 2.6.2. The effect of modulating these parameters is visualized in the frequency-current (FI) curves shown in Figure 3, where threshold modulation (Figure 3A) shift the FI response and time constant modulation (Figure 3B) changes the shape of the response curve.

### 2.3 Gaussian perturbation simulation

We simulate two forms of perturbation to the weight values, and evaluate the ability of the context-dependent adaptation to recover the loss of accuracy that would otherwise result from these weight perturbations in the absence of any adaptation. Gaussian perturbations )φ∈N(0,1)) were added to the learned weights of the convolution layers and one of the linear layers. Each sample from the standard normal was scaled by the weight value **w** and the context input, as described by Equation 6:


$$w_{perturbed}=w+c|w|\cdot \varphi$$ (6)

**Figure 4:**
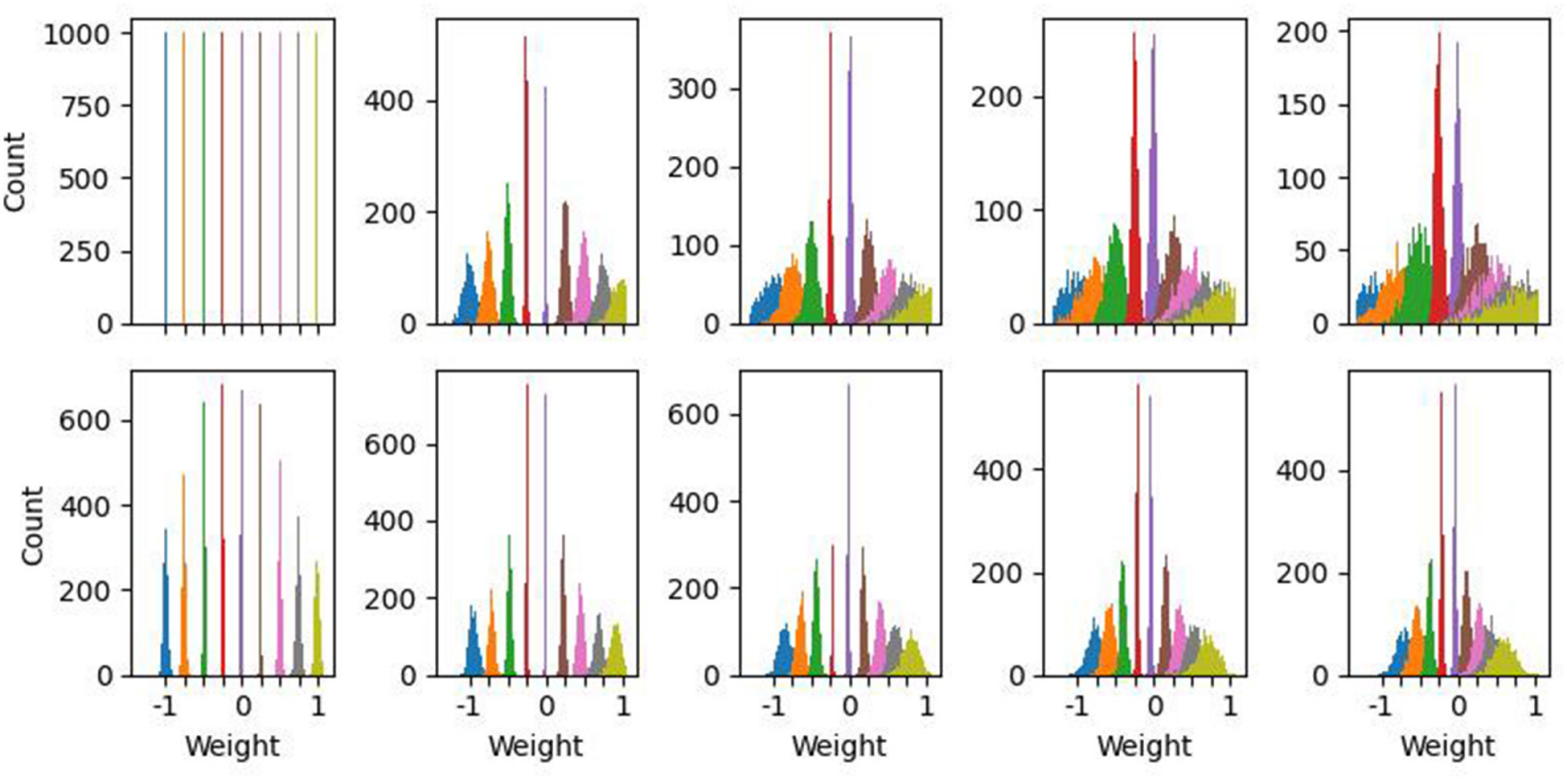
Effect of weight perturbations under both the Gaussian and TID mechanisms. X-tick locations indicate desired weights, and colored counts indicate distributions around a target weight. **(Top)** Effect of increasing Gaussian perturbations on the weight distributions. Note that as perturbation level increases, the spread, but not mean, of the weights increases. **(Bottom)** Effect of TID on weights stored in SONOS devices. The zero-context (far-left) has non-ideal weights due to intrinsic write errors in the devices. These non-idealities increase further as context (TID) increases. Compared to the Gaussian sweep, TID has a smaller effect on spread, but comes with an average change in weight compared to the target value.

The perturbation was randomly initialized on each trial, but held constant over the time steps within a trial. The perturbed weights therefore represent the slowly-varying random perturbations discussed in the introduction, rather than the fast intrinsic stochasticity of synaptic release. The context *c* modulates the level of perturbation proportional to the magnitude of the trained weight **w**. To allow a systematic evaluation the effect of different perturbation levels, we utilize 10 discrete context values, evenly spaced across [0, 1.0], representing weight-specific perturbation in the form of Gaussian distributions with standard deviation in the same range. The increase in the Gaussian perturbation-level has the effect of increasing the spread but not the mean of the weight distribution as shown in Figure 4 (Top).

### 2.4 SONOS ionizing radiation simulation

We also tested our adaptive network algorithms on a more realistic and complex form of device conductance error that is induced by a harsh operating environment. As an exemplar, we assume that the programmable resistors that implement the weights of the convolutional and linear layers are SONOS (silicon-oxide-nitride-oxide-silicon) charge-trap memory devices, and that the entire system is exposed to ionizing radiation, for example, X-rays, gamma rays, or protons that may be encountered in space. The cumulative amount of ionizing radiation that is delivered to the system is the total ionizing dose (TID); the TID starts at zero and increases over time, and this is used as the context signal *c*. In a practical system, the TID can be independently measured using a dosimeter and this value can be passed to the neuron circuits. Ionizing radiation perturbs the amount of charge that is stored in the SONOS devices, and the induced change in SONOS conductance with TID has been experimentally characterized in a prior work (Xiao et al., 2024). For a particular weight *w* that is stored in SONOS, the effect of TID is to add a perturbation N[μ(w,TID),σ(w,TID)]. Here, μ describes the deterministic (or mean) component of the perturbation, σ describes the random component, and the empirical dependencies on *w* and TID are obtained from (Xiao et al. 2024). Figure 4 (Bottom) summarizes the effect of ionizing radiation on the individual weights stored in SONOS devices, as a function of TID. Therefore, for the case of ionizing radiation, the induced error increases with the context signal (TID) but changes in a more complicated and arbitrary way compared to simple Gaussian noise. We choose 10 equally spaced context levels corresponding to TID values from 0 to 1,000 krad in 100 krad steps.

### 2.5 Model variations

To test the effectiveness of the context-dependent adaptation, we consider four different model designs as described in Table 2.

**Table 2:** Model descriptions.

**Modulation**	**Perturbation**	
	**Absent**	**Present**
Absent	Base	Perturbed
Present	Sham	Context

The *base model* implements the network architectures described above, without perturbations added to the weights or the context-dependent adaptations described in Equations 4, 5. It acts as the overall baseline, indicating how well the chosen network architectures perform on the exemplar tasks. The *perturbed model* implements the weight perturbation as described in Equation 6, but does not provide the adaptive mechanism. It demonstrates how the performance degrades with increased perturbations. The *sham model* includes the adaptive mechanism, but has no perturbation added to the weights. This acts as a control to determine if the network achieves improved performance solely from having additional trainable parameters. These three models are compared to the *context model*, which includes the perturbed weights and the adaptive mechanism. The degree to which the context model improves over the perturbed model, toward the baseline performance, is indicative of the network performance recovering from the effects of perturbations through the adaptive parameters.

### 2.6 Tasks and training

#### 2.6.1 Tasks

Models were evaluated on two tasks: Image Classification (CIFAR-10) (Krizhevsky, 2009) and a Small Object Tracking (SOT) Task, which is temporal in nature (Chapman et al., 2024). In each case, testing datasets were separate from training, and all reported performance metrics are based on the testing set, averaged across 10 independent initializations. CIFAR-10 images were presented for 32 timesteps along with the context input, and the network prediction was based on activity in the final timestep, with a loss function of cross-entropy and performance reported as top-1 classification accuracy. In the SOT task, videos consisted of 100-timesteps of a single-pixel object moving over a background, similar to what would be observed in a remote-sensing application. SOT predictions are (x,y) coordinates of the object location on each timestep, with both loss and performance measured as mean-square error (MSE).

#### 2.6.2 Hyperparameters and setup

Except where noted, hyperparameters and initializations followed PyTorch Lightning (Falcon and The PyTorch Lightning team, 2019) or Norse (Pehle and Pedersen, 2021) defaults. Training used backpropagation through time, combined with stochastic gradient descent with a momentum optimizer (Kingma and Ba, 2017) for weights and using the SuperSpike (Esser et al., 2016) surrogate gradient applied to spiking activations. The learning rate was initialized at 0.001 and decreased by a factor of 0.5 whenever the model performance stopped improving over 10 epochs. A parameter sweep on the membrane time constant τ_m_ (Equation 1) was performed for the base feedforward and recurrent models for the adaptive threshold experiments. Optimal values were determined to be four frames and 16 frames, respectively. These values were used for all model variations of the feedforward and recurrent networks. All training was performed on a single compute node with 512GB DDR4 memory and an NVIDIA A100 (40GB) GPU.

#### 2.6.3 Pretraining

All models were pretrained without perturbations or context-dependent mechanisms (base model) for 100 epochs. This process allows the weights to converge so the network can perform the task under ideal conditions. Afterwards, each model variation described in Table 2 was trained for an additional 100 epochs. During this phase, models with the context-dependent adaptation (Sham and Context Models) trained the threshold or time-constant (parameter **p** in Equation 4 or Equation 5) in addition to the weights. Models without the context-dependent adaptation continued to train weights in the absence (Base) or presence of perturbations.

### 2.7 Train-test tradeoff

In order to thoroughly investigate the tradeoff of perturbation-tolerance vs. performance at low-perturbation levels, we performed all pairwise combinations of training and testing perturbation levels for the CIFAR-10 networks. For each model variation (Table 2), network topology (feedforward/recurrent), and context-dependent adaptation, we trained the models to a maximum of each of the 10 discrete context values (perturbation levels) described above. Each model was then tested on a hold-out test dataset with weight perturbations applied at each level.

### 2.8 Latent space analysis

To further investigate the mechanism through which adaptive parameters counteract synaptic perturbations, we utilized principal component analysis (PCA) to visualize latent space activations (Ságodi et al., 2023). We recorded all sub-threshold membrane potentials of the penultimate activation layer networks during inference, and performed per-trial PCA using scikit-learn (Pedregosa et al., 2011) on a subset of the CIFAR-10 classes, and then visualized per-class for baseline, perturbed, and context networks, to observe the effect of perturbations and adaptive terms on network activity. We used the perturbed and context networks trained up to the highest perturbation level (1) for the analysis and ran inference on a subset of images with three different context values: 0, 0.2, and 1.

## 3 Results

### 3.1 Gaussian perturbation models

**Figure 5:**
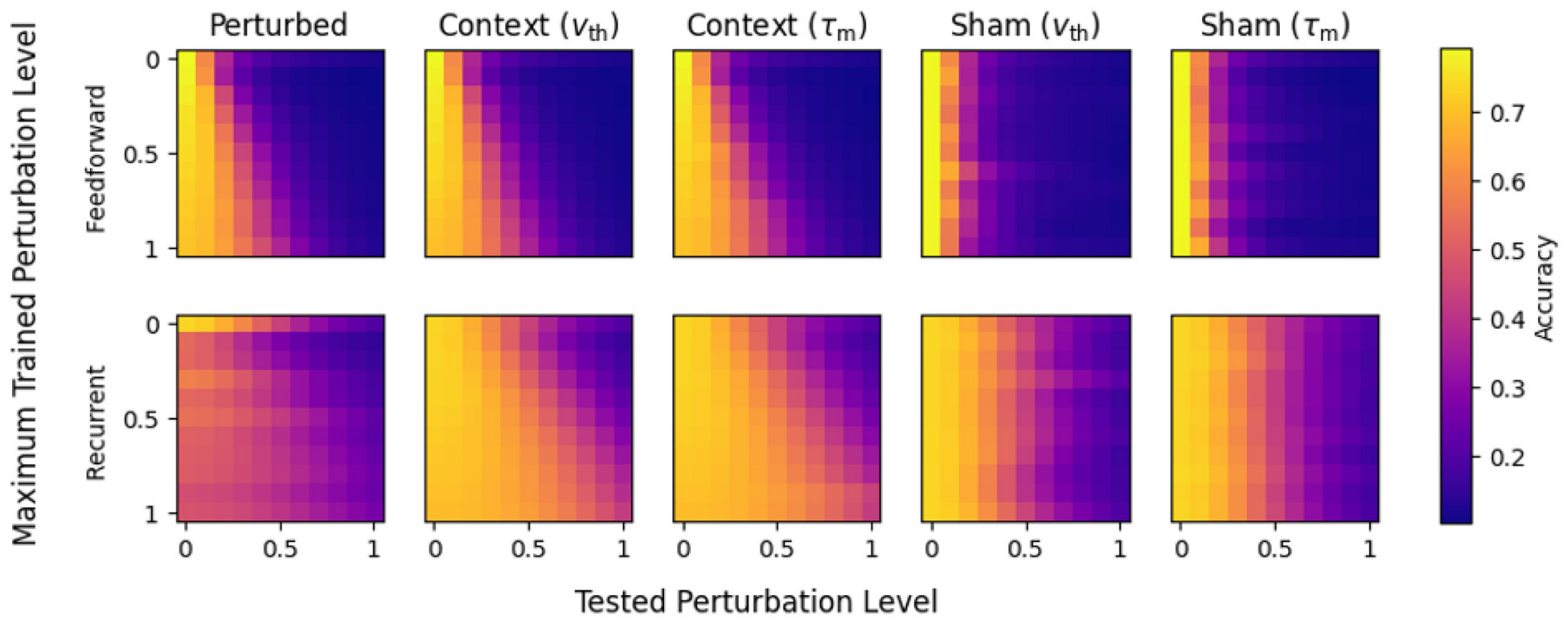
Accuracy of each network for each model and parameter type (*v*_th_ or τ_m_). The two rows show results for either feedforward or recurrent network topologies, while the five columns show different models (perturbed, context and sham), defined in Table 2. Note that the base model results are presented in the first row of each image for comparison. For each network and model variation, the maximum trained perturbation level varies over the y-axis, and the model is separately evaluated on each perturbation level (*x*-axis). (Perturbed, **Top**) The feedforward perturbed model shows a general trend of highest performance when tested with no perturbations. Models with higher training perturbations are resilient to higher test perturbations, but still have significantly degraded performance. (Context, **Top**) Context-modulated feedforward networks show a general trend similar to the perturbed model, indicating minimal-to-no benefit from the context mechanisms. (Sham, **Top**) The sham performed roughly the same as the baseline under all tested perturbation levels (first row is the base model), indicating the adaptive parameters were not helpful as additional trained parameters without exposure to perturbations during training. (Perturbed, **Bottom**) Compared to feedforward models, the recurrent models show a slower drop off in performance as the tested perturbation level increases (horizontally). (Context, **Bottom**) Aside from the lowest levels of perturbation, the recurrent networks with either adaptive mechanism have higher accuracy for the same level of trained/tested perturbation as feedforward models. Similar to the feedforward network, the models with higher maximum trained perturbation levels were resilient to higher test perturbations, but having the additional adaptive mechanism slowed the reduction in performance substantially more in the recurrent network as the perturbation level increased. (Sham, **Bottom**) Similar to the feedforward, the sham model performed roughly the same as the base model under all tested perturbation levels, but shows more graceful degradation of performance with increasing test perturbation levels compared to the feedforward model.

Figure 5 shows the performance of all network configurations with Gaussian weight-perturbations in the CIFAR-10 task. Each panel shows the response of a specific network configuration, trained under a different maximum perturbation (*x*-axis) and tested in specific perturbation levels (*y*-axis). In feedforward networks (top) the perturbed model showed a trend of performing better for moderate perturbation levels when trained at high levels of perturbation, but at the cost of low-perturbation performance, and never achieved greater than 50% accuracy for test perturbation levels above 0.3. Neither method of context-dependent adaptation was able to significantly improve this performance for feedforward networks. Feedforward sham networks showed high performance only under conditions without perturbations. Overall, these results indicate that feedforward networks degrade quickly with increasing levels of perturbation, and do not benefit from context-dependent adaptive parameters.

In contrast, recurrent configurations (Figure 5, bottom) showed strong responses to adaptive parameters. The perturbed model performs overall worse than baseline, but degrades smoothly as the tested perturbation level is increased, across all training levels. Both adaptive threshold and adaptive time-constant networks showed significant increase in performance over the perturbed model. In general, models that were trained with higher perturbation distributions retained accuracy at higher levels of test perturbations. Notably, in no case did training to higher levels of perturbations decrease performance for a given test perturbation level (columns of individual panels), indicating that training is tolerant to high perturbation levels and preserves performance for low levels when context signals are provided. The recurrent sham models both show a smoother degradation of performance as the tested perturbation level increases, compared to feedforward sham models. However, these models perform on-par with the base models, indicating that having additional trainable parameters introduced by the context-dependent adaptation does not increase overall performance when not exposed to perturbations.

### 3.2 Mechanisms of adaptation

To evaluate the mechanism by which adaptation counteracts perturbation-induced inaccuracies, we evaluated the learned adaptive components of neural dynamics, and the effect that these parameters had on neural activity.

#### 3.2.1 Learned adaptive parameters

**Figure 6:**
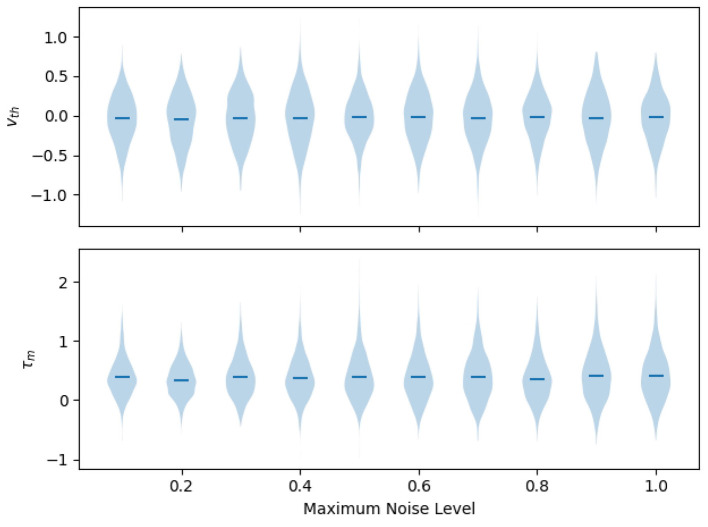
Distribution of adaptive projection vectors in fully trained networks. The *x*-axis indicates the maximum level of perturbation the network was trained under. **(Top)** In threshold-modulated networks, thresholds are increased both positively and negatively, without a significant non-zero mean. Distributions are not significantly different across networks trained at different perturbation levels. **(Bottom)** In adaptive time-constant networks, the adaptive portion of the inverse time constant is significantly greater than zero, indicating that the network is primarily sped-up compared to baseline models. There is a significant, but low magnitude, increase in modulation in networks trained to high levels compared to low-perturbation training.

First, we evaluate the learned adaptive parameters for both threshold modulated and time-constant modulated recurrent networks that were trained to various maximum levels of perturbations, as summarized in Figure 6. Threshold voltages do not have a significantly non-zero mean (*t*-test; *p*>0.05, Bonferroni corrected), and we found no significant difference in distributions across perturbation-training levels (Kolmogorov–Smirnov test, *p*>0.05, Bonferroni corrected for multiple comparisons). Time constants were significantly non-zero for each perturbation-trained level (*t*-test; mean = 0.386 frames, *p* < 0.05, Bonferroni corrected). There were significant pairwise differences between *c* ≤ 0.2 and *c*≥0.8 (Kolmogorov–Smirnov; mean = 0.389 frames for *c* = 0.1, 0.41 frames for *c* = 1.0, *p* ≤ 0.001, Bonferroni corrected). Overall, these results suggest that time-constant modulation has a net slowing effect, as indicated by a mean-positive increase, while threshold modulation is more distributed in nature. Despite these differences, Figure 5 demonstrates that both mechanisms achieve the same overall performance, suggesting additional mechanisms at the network-activity level, rather than solely by modulating individual neuron responses.

#### 3.2.2 Latent space analysis

**Figure 7:**
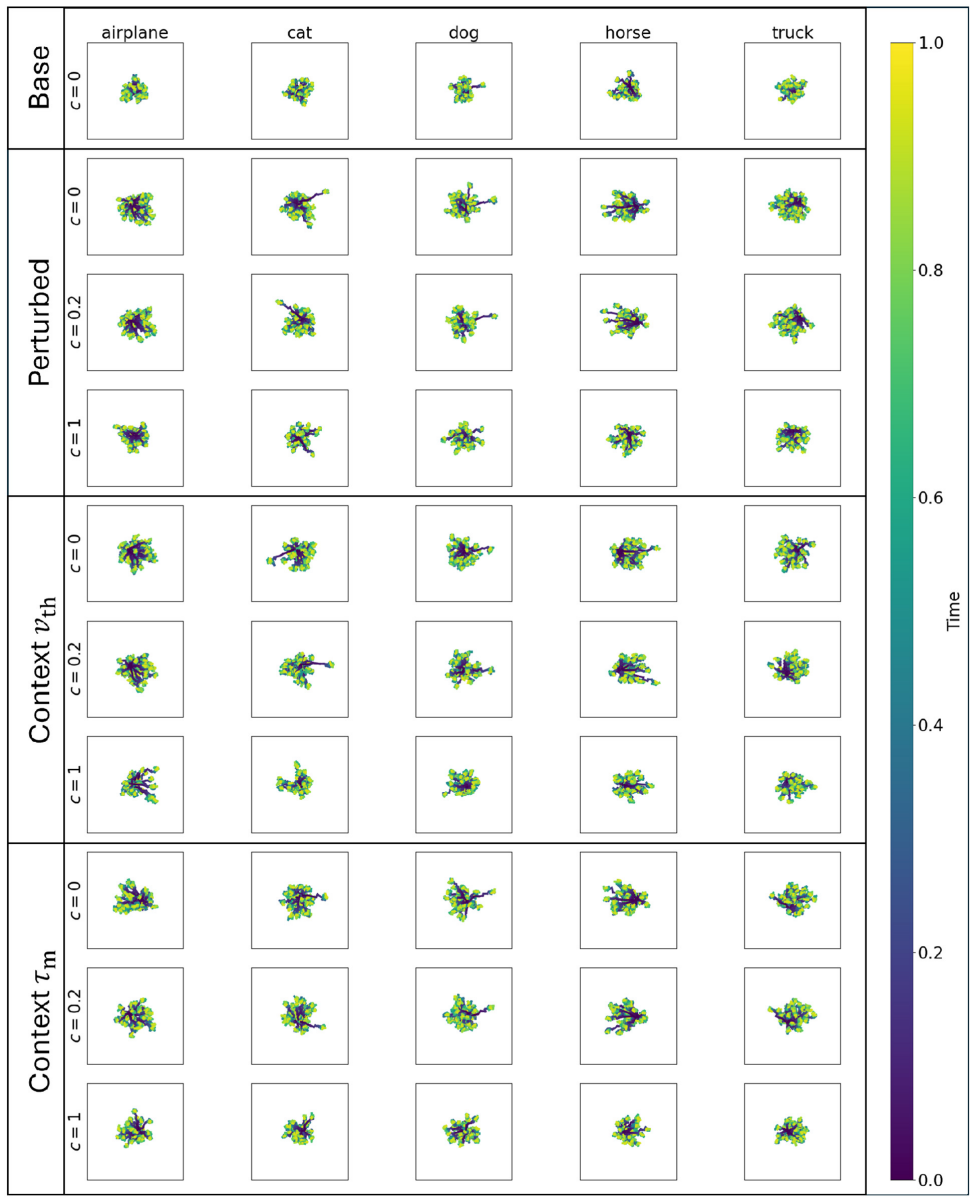
Latent representations for baseline, perturbation-trained, and adaptive threshold and time-constant models for the feedforward network, showing the evolution of the first two principal components for 50 representative trials (32 time steps in each trajectory) for five of the CIFAR-10 classes. Base models begin (blue) near the origin and move slightly in both principal components. Compared to recurrent models (Figure 8), there is greater intra-class variability in final location (yellow). Perturbed models tend to move further from origin, but without a cohesive movement direction. Context models behave similarly to perturbed models.

To evaluate how the zero-mean threshold or small time-constant adaptive parameters described above effected network performance, we investigated latent space representations of the context models compared to baseline and perturbed models. Figure 7 shows the results for feedforward networks, where each panel shows the first two principal components for a subset of 50 images from five of the CIFAR-10 classes. Each image was presented to the network over 32 time steps. Feedforward networks showed a radially symmetric movement in both components, with no linearly separable differences between classes. As expected from the performance results discussed above, the perturbed and context models show similar behavior.

**Figure 8:**
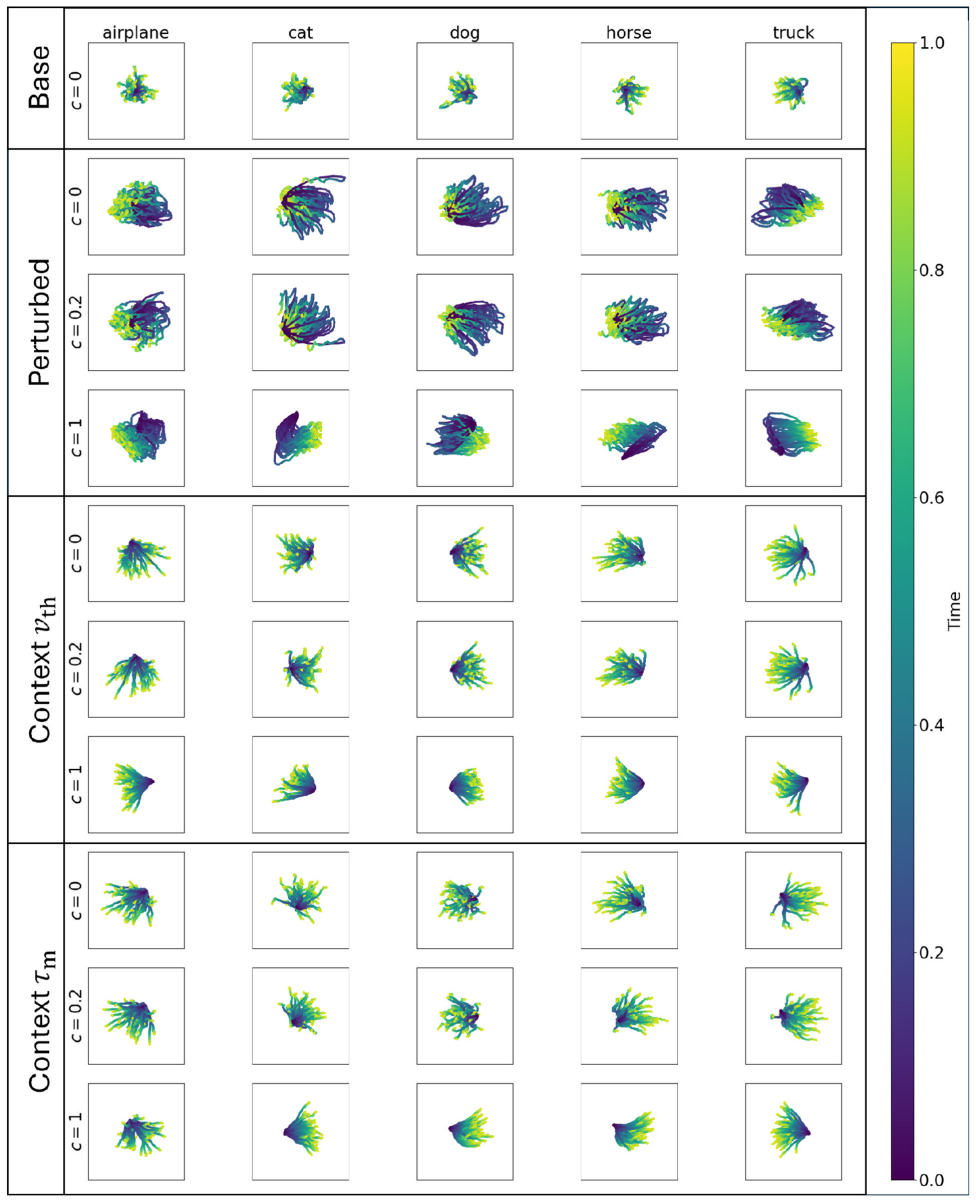
Latent representations for recurrent neural networks, similar to the Feedforward results in Figure 7. Base models have a unique final (yellow) location for different classes, and individual trajectories move to the final location over the 32 time steps. Perturbed models tend to start with a wider range of initial locations, and wider range of positions over time, but converge to similar final locations across stimuli. Critically, as perturbation levels increase, the final PCA location changes within class. Threshold Context Models with context have different final PCA locations, similar to baseline models, but the magnitude of these locations is larger than the baseline. Trajectories start near the origin, and move smoothly to the final location, with similar dynamics across perturbation levels. Time-constant Context Models have a similar behavior to threshold modulated networks, despite the difference in underlying single-neuron response to contextual signals.

In contrast to the feedforward activities, the recurrent network activities of Figure 8 showed strong differences between baseline, perturbed, and context-modulated networks. The baseline trajectories show a smooth progression from the PCA-origin from the first time step to a final region which varies for each class. Conversely, perturbed models had less smooth trajectories, and the final location within each class changed as the perturbation level increased. This suggests that the network activity, for identical stimuli, could result in different final locations as the perturbation level increases, and that the separation of class trajectories is difficult compared to the baseline condition. Counterintuitively, the perturbed models were smoother at higher perturbation levels, suggesting that the network is primarily driven by task-irrelevant activity. Threshold modulated networks recovered the uniqueness of the trajectories observed in the base model and approached similar locations across perturbation levels, but had larger magnitudes in the final locations compared to the base model. The recurrent adaptive time-constant model showed similar trends as the adaptive threshold model. Notably, despite the lower change in parameter values compared to threshold, the magnitude of the PC locations for time-constant and threshold models is similar. Overall, these results show that both adaptive mechanisms recover not only the performance but also network-level dynamics of the recurrent baseline models.

### 3.3 Device perturbation models

**Figure 9:**
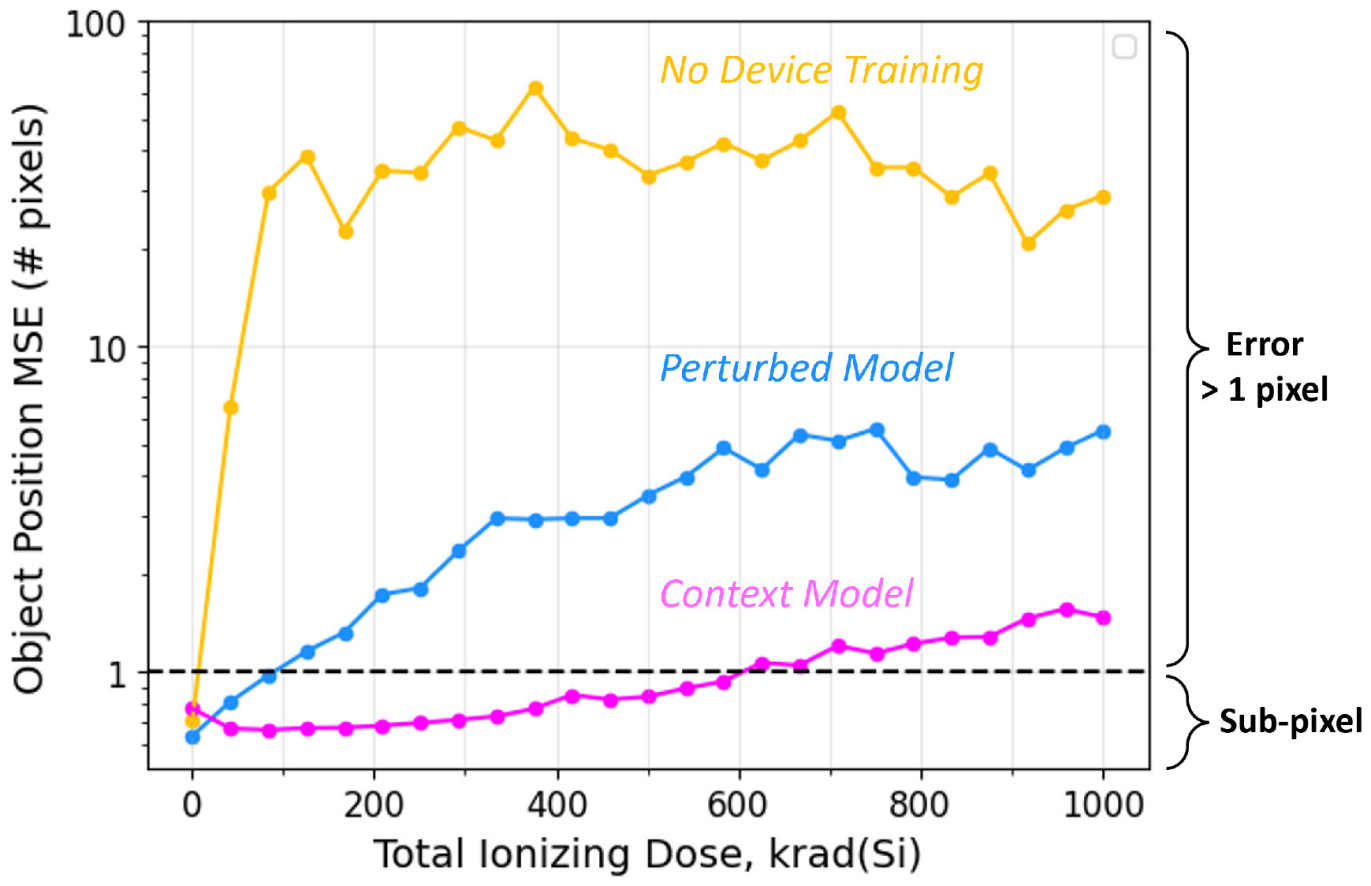
Model performance on the small-object tracking task. Yellow: With no hardware-aware training performs poorly for even small levels of perturbation. Blue: When trained with perturbed weights at all levels, but no context mechanism the model performs well at low levels of perturbation without affecting the performance under ideal conditions. Magenta: The context model performs with an error of less than one pixel, up to moderate levels (600 krad).

As a more realistic edge-computing scenario, we evaluate the same adaptive parameter approaches from above to a small object tracking remote sensing task, by simulating the effects of ionizing radiation on SONOS device conductances. Figure 9 summarizes the results of this experiment. The device agnostic training (Figure 9, yellow) shows the error when the model is trained without radiation effects and tested with radiation effects. This model is accurate to sub-pixel (mean-square error less than one pixel) tracking only in the absence of any ionizing radiation (which results in weight distributions from the bottom left panel of Figure 4) that have very low errors. When the TID increases even slightly, however, the error quickly increases to near-chance levels, showing that this network is less robust to weight perturbations than the CIFAR10 experiments. The perturbed model (Figure 9, cyan) is trained across all TID levels, without any context-dependent adaptation, and shows tolerance to low-levels of weight perturbations (up to 100 krad). The context models (Figure 9, magenta) however show sub-pixel accuracy up to 600 krad, with less than 2-pixels of error up to the highest level of radiation tested.

## 4 Discussion

Here we show that a global scalar-valued context signal can act as a modulator to allow recurrent neural networks to achieve high accuracy across a wide range of perturbations in synaptic weights. This is achieved through the training of a neuron-specific adaptive parameter, in the form of neural parameters such as the voltage threshold or membrane time-constant.

We found that the context-dependent adaptation was able to restore performance in recurrent, but not feedforward networks. This is supported by other theoretical work which demonstrates that recurrent neural networks can dynamically switch input-output relationships as a function of context-like signals (Ferrand et al., 2023; Baronig and Legenstein, 2023). We also see that performance degrades more gracefully in recurrent networks (Ságodi et al., 2023), even in the absence of the adaptive mechanism. Theoretical work has argued that recurrent, but not feedforward, neural networks perform dynamic mapping of stimuli into latent representations (Pellegrino and Chadwick, 2025). By this argument, the context-dependent adaptation mechanisms investigated here may recover dynamic mappings that would otherwise be disrupted in perturbed scenarios.

Further investigating the learned adaptive parameters and latent activities, we found that the population-level adaptation was different in threshold vs. time-constant-modulated networks. The adaptive threshold networks showed a zero-centered distribution in dynamic membrane potential. While this effect was therefore not overall excitatory or inhibitory, the magnitude of change in threshold was larger than time-constant modulation was. In contrast, the adaptive time-constant had a net slowing effect on the neural populations, but corresponded to a lower proportional change relative to baseline. These results support theoretical and mathematical modeling of decision making processes which posit that decisions are slowed in the presence of global uncertainty (Shadlen and Newsome, 2001). More recent theoretical work (Islah et al., 2025) has theorized that context-like modulation, in the form of distal dendritic inputs, is responsible for this uncertainty-driven behavioral difference. Such speculation is further supported by causal experimental evidence of multi-region control of decision making under uncertainty (Yartsev et al., 2018; DePasquale et al., 2024).

While the neural-level projection vector for the adaptive threshold was zero-centered, the population level PCA shows that the latent space representations are slowed and stabilized by the adaptive threshold, and the adaptive time-constant resulted in the same overall network dynamics as the adaptive threshold despite having a slowing effect on the individual neural dynamics. Beyond overall slowing of representation-space activity, we observed that the learned adaptive parameters, at the population level, were able to restore the direction of activity, demonstrating that learned population projections can be more powerful than a global increase in certainty required for a decision (Summerfield and De Lange, 2014).

Taken together, our results highlight that this adaptive mechanism is best understood through a dynamical-systems lens. In recurrent networks, context-dependent modulation reshapes the underlying state-space flow. This allows the network to recover appropriate trajectories and restore task-relevant latent dynamics after perturbation. Feedforward architectures however lack the internal state and attractor-like structure needed for dynamical remapping and trajectory repair. This conclusion is consistent with prior work showing that context-like signals enable flexible reconfiguration of recurrent dynamics (Ferrand et al., 2023; Baronig and Legenstein, 2023; Pellegrino and Chadwick, 2025).

The decision-like processing described above assumes a global uncertainty signal which corresponds to short-timescale uncertainty in stimuli. However, the weight drift that is induced by environmental exposure such as the studied TID effect is more similar to phenomena such as representational drift. During this drift, the variables and parameters of the network change over time such that the activity of the neurons and network changes (Driscoll et al., 2022; Geva et al., 2023). The brain is able to compensate for this drift so that the performance of specific tasks does not degrade overtime despite the changing network. In addition to ongoing changes in biological variability, in theory, the drift occurs because the brain is not only optimizing for a single task, but, continually adapting to maximize performance on many different tasks. With respect to one task, the network variables such as the synaptic weights appear perturbed. In longer timescale scenarios the context signal may arise from prediction errors, which indicate that previously learned associations are uncertain (Hertäg et al., 2025), or distance in episodic memory as measured by hippocampal representations (Climer et al., 2025; Geva et al., 2023).

As discussed in Section 1, there are several possible mechanisms by which biological systems may implement global context modulation. Methods include the multi-compartment approaches of (Baronig and Legenstein 2023) and (Ferrand et al. 2023). In this work we chose to use the additive approach of (Helfer et al. 2023) to modulate the voltage threshold and membrane time constant, but future work may involve exploring the other approaches to determine if having explicit apical compartments included in the neuron model could have benefits over the chosen approach. Other mechanisms such as neuro-modulation may provide more detailed mechanisms which affect combinations of membrane dynamics, firing thresholds, leak voltages, and other properties. Such detailed biological mechanisms may allow for multiple independent context-modulated responses within a single neuron.

From a practical neuromorphic engineering perspective, our biologically motivated investigation of context-modulated adaptation demonstrates how spiking neural networks may overcome inherent perturbations in weights implemented in analog approaches. While analog artificial neural networks are highly energy efficient compared to purely digital approaches, they have tolerance only to low-levels of weight perturbations and have relatively high energy consumption in analog-to-digital conversion. The base leaky-integrate and fire model we utilize here has been implemented in CMOS devices with substantially lower energy consumption than standard ADCs typically used with analog weights (Indiveri et al., 2011). The modifications required to implement neuron-specific adaptive parameters, as illustrated in Figure 1B are compatible with previous analog implementations of LIF neurons (Garg et al., 2024; Oli-Uz-Zaman et al., 2022), allowing for only minor modification and increase in hardware size compared to these previous approaches. Easy implementation of our context-adaptive parameters to existing implementations.

Overall, this work demonstrates a simple mechanism by which global context signals, which correspond to perturbation levels in analog neuromorphic platforms, can be combined with neuron-specific mechanisms for adaptive edge-based computing platforms.

## Data Availability

The original contributions presented in the study are included in the article/supplementary material, further inquiries can be directed to the corresponding authors.

## Author disclaimer

Any subjective views or opinions that might be expressed in the written work do not necessarily represent the views of the U.S. Government. The publisher acknowledges that the U.S. Government retains a non-exclusive, paid-up, irrevocable, world-wide license to publish or reproduce the published form of this written work or allow others to do so, for U.S. Government purposes. The DOE will provide public access to results of federally sponsored research in accordance with the DOE Public Access Plan.
